# Supplementary material for: Large-scale discovery of protein interactions at residue resolution using co-evolution calculated from genomic sequences
Source: Nat Commun. 2021 Mar 2;12:1396. doi: 10.1038/s41467-021-21636-z (PMC7925567; doi:10.1038/s41467-021-21636-z)
Supplement: Supplementary file 13 — Supplementary Data 11 [file 41467_2021_21636_MOESM13_ESM.zip › supplementary_prediction/pe_vs_inner_mem011299_DEGS_LNT/pe_vs_inner_mem011299_DEGS_LNT.pdf]

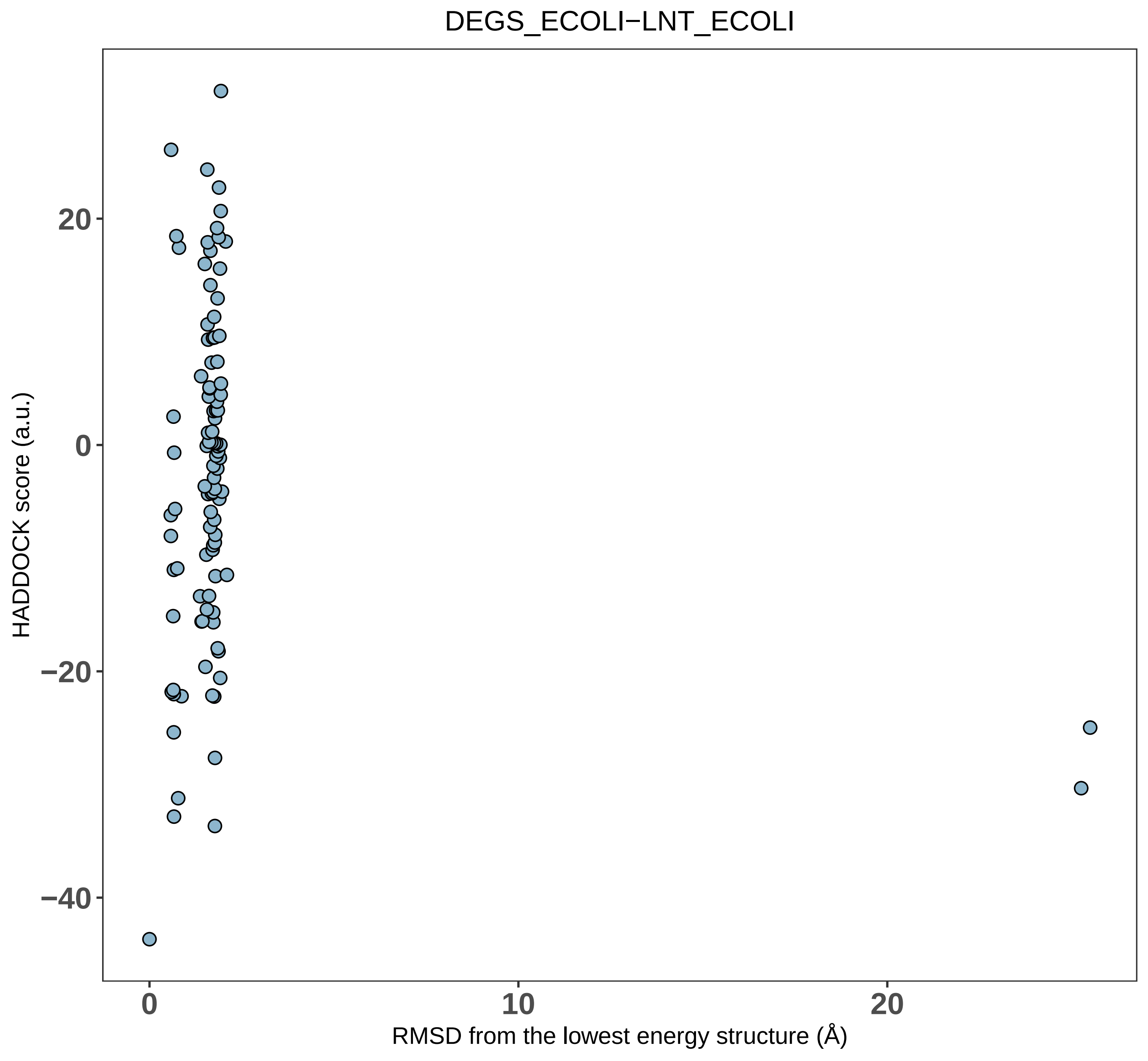
DEGS_ECOLI−LNT_ECOLI
HADDOCK score (a.u.)
20
0
−20
−40
0
10
20
RMSD from the lowest energy structure (Å)
